# ^99m^Tc-CXCR4-L for Imaging of the Chemokine-4 Receptor Associated with Brain Tumor Invasiveness: Biokinetics, Radiation Dosimetry, and Proof of Concept in Humans

**DOI:** 10.1155/2020/2525037

**Published:** 2020-04-27

**Authors:** Paola Vallejo-Armenta, Clara Santos-Cuevas, Juan Soto-Andonaegui, Rosa M. Villanueva-Pérez, Jorge I. González-Díaz, Francisco O. García-Pérez, Angélica Arrellano-Zarate, Myrna Luna-Gutiérrez, Erika Azorín-Vega, Blanca Ocampo-García, Guillermina Ferro-Flores

**Affiliations:** ^1^Departamento de Medicina Nuclear, Hospital de Especialidades del Centro Médico Nacional Siglo XXI, Ciudad de México 06720, Mexico; ^2^Departamento de Materiales Radiactivos, Instituto Nacional de Investigaciones Nucleares (ININ), Ocoyoacac 52750, Estado de México, Mexico; ^3^Departamento de Medicina Nuclear, Instituto Nacional de Cancerología, Ciudad de México 14000, Mexico

## Abstract

Overexpression of the chemokine-4 receptor (CXCR4) in brain tumors is associated with high cancer cell invasiveness. Recently, we reported the preclinical evaluation of ^99m^Tc-CXCR4-L (cyclo-D-Tyr-D-[NMe]Orn[EDDA-^99m^Tc-6-hydrazinylnicotinyl]-Arg-NaI-Gly) as a SPECT radioligand capable of specifically detecting the CXCR4 protein. This research aimed to estimate the biokinetic behavior and radiation dosimetry of ^99m^Tc-CXCR4-L in healthy subjects, as well as to correlate the radiotracer uptake by brain tumors in patients, with the histological grade of differentiation and CXCR4 expression evaluated by immunohistochemistry. ^99m^Tc-CXCR4-L was obtained from freeze-dried kits prepared under GMP conditions (radiochemical purities >97%). Whole-body scans from six healthy volunteers were acquired at 0.3, 1, 2, 4, 6, and 24 h after ^99m^Tc-CXCR4-L administration (0.37 GBq). Time-activity curves of different source organs were obtained from the image sequence to adjust the biokinetic models. The OLINDA/EXM code was employed to calculate the equivalent and effective radiation doses. Nine patients with evidence of brain tumor injury (6 primaries and 3 recurrent), determined by MRI, underwent cerebral SPECT at 3 h after administration of ^99m^Tc-CXCR4-L (0.74 GBq). Data were expressed as a *T*/*B* (tumor uptake/background) ratio. Biopsy examinations included histological grading and anti-CXCR4 immunohistochemistry. Results showed a fast blood activity clearance (*T*_1/2_*α* = 0.81 min and *T*_1/2_*β* = 12.19 min) with renal and hepatobiliary elimination. The average equivalent doses were 6.10*E* − 04, 1.41*E* − 04, and 3.13*E* − 05 mSv/MBq for the intestine, liver, and kidney, respectively. The effective dose was 3.92*E* − 03 mSv/MBq. SPECT was positive in 7/9 patients diagnosed as grade II oligodendroglioma (two patients), grade IV glioblastoma (two patients), grade IV gliosarcoma (one patient), metastasis, and diffuse astrocytoma with *T*/*B* ratios of 1.3, 2.3, 13, 7, 19, 5.5, and 3.9, respectively, all of them with positive immunohistochemistry. A direct relationship between the grade of differentiation and the expression of CXCR4 was found. The two negative SPECT studies showed negative immunohistochemistry with a diagnosis of reactive gliosis. This “proof-of-concept” research warrants further clinical studies to establish the usefulness of ^99m^Tc-CXCR4-L in the diagnosis and prognosis of brain tumors.

## 1. Introduction

The aggressiveness of high-grade glioblastomas and the refractoriness to conventional therapies are mostly due to their highly invasive nature [1]. Glioblastomas produce many malignant tissue satellites that frequently migrate to substantial distance from the primary tumors, the reason for which they are challenging to eradicate by surgical techniques and chemo- and radiotherapeutic regimens [2, 3].

Previous studies have demonstrated that the overexpression of the chemokine-4 receptor (CXCR4) results in increased migration of glioma tumor cells [4]. This expression is from twenty-five to eighty-nine times higher than that found in noninvasive (low-grade) glioma cells [5]. High expression levels of CXCR4 and its ligand, the chemokine stromal cell-derived factor 1-α (SDF1-*α* = CXCL12), usually indicate a poor prognosis for patients with brain tumors [6]. Therefore, CXCR4 and CXCL12 are molecular targets of interest for the development of potential targeted cancer therapies.

Recently, a ^68^Ga-labeled cyclic DOTA-pentapeptide (^68^Ga-Pentixafor®) was reported as a successful positron emission tomography (PET) tracer, useful for visualization of CXCR4 expression in patients with glioblastoma [7]. Preclinical studies of *N*-[^11^C]methyl-AMD3465 PET ligand have also demonstrated the feasibility of obtaining *in vivo* images of CXCR4 expression in glioma tumors [8]. Both PET tracers might be useful in identifying patients to whom potential CXCR4-targeted radiotherapies (e.g., ^177^Lu-CXCR4-ligands) could be beneficial [9, 10]. Other CXCR4-ligands for PET and SPECT have also been reported for the detection of CXCR4 expression in different tumors, but only at a preclinical stage [11–14]. ^68^Ga-Pentixafor® is the only CXCR4-ligand used in clinical protocols.

Our group recently reported the synthesis, formulation, and preclinical evaluation of ^99m^Tc-CXCR4-L (cyclo-D-Tyr-D-[NMe]Orn[EDDA-^99m^Tc-6-hydrazinylnicotinyl]-Arg-NaI-Gly) as a SPECT radioligand capable of specifically detecting the CXCR4 protein [9].

This research aimed to estimate the biokinetic behavior and radiation dosimetry of ^99m^Tc-CXCR4-L in healthy subjects, as well as to correlate the radiotracer uptake by brain tumors in patients, with the histological grade of differentiation and CXCR4 expression evaluated by immunohistochemistry.

## 2. Materials and Methods

### 2.1. Reagents

A CXCR4-L (cyclo-D-Tyr-D-[NMe]Orn[6-hydrazinylnicotinyl]-Arg-NaI-Gly, MW 837 g/mol) freeze-dried kit formulation was obtained from the “Instituto Nacional de Investigaciones Nucleares” (ININ, Mexico) prepared under GMP conditions [9]. ^99m^TcO_4_Na was obtained from a GETEC ^99^Mo/^99m^Tc generator (ININ, Mexico). All the other reagents were used as received from Sigma-Aldrich Chemical Co.

### 2.2. Preparation of ^99m^Tc-CXCR4-L

Technetium-99m-labeled CXCR4-L (^99m^Tc-CXCR4-L) was prepared according to the supplier's instructions [9]. In brief, radiolabeling was achieved by adding 1 mL of 0.2 M phosphate buffer (pH 7.0) solution to the CXCR4-L lyophilized formulation (GMP-grade, ININ-México) with immediate addition of 0.37–0.74 GBq (1 mL) of ^99m^TcO_4_Na eluant and incubation at 95°C in a dry bath (heating block) for 10 min. The radiochemical purities of ^99m^Tc-CXCR4-L were assessed by reverse-phase radio-HPLC as reported by Avila-Sanchez et al. [9].

### 2.3. Healthy Subjects and Patients

Six healthy subjects (age range: 28–37 y; mean age ± SD: 33 ± 4 y; 3 females and 3 males) were included. Physical examination and medical history were performed. Individuals with a history of major surgery (e.g., organ removal) or evidence of clinical disease were excluded. Mean (±SD) subject weight was 62 ± 10 kg (range, 51–76 kg). After receiving full information concerning the aims of the study, all volunteers agreed to participate in the complete biokinetic study and signed a consent form. The activity administered to healthy subjects was 0.37 GBq (25 *μ*g of CXCR4-L peptide).

Nine patients between 25 and 72 y (mean age ± SD: 42 ± 16 y), with evidence of brain tumor injury (6 with an indeterminate brain tumor and 3 with suspicion of tumor recurrence), established by magnetic resonance imaging (MRI) studies, were included in the study (Table 1). This research was carried out at the Nuclear Medicine Department at the Specialties Hospital of “Centro Médico Nacional Siglo XXI, Instituto Mexicano del Seguro Social,” Mexico. Informed consent was given by the patients, and the protocol was approved by the institutional ethics committee, considering the following aspects: (a) Helsinki Declaration (1975; revised version, 2008) and the ethical standards of the institutional committee related to human experimentation, (b) the GMP certificate granted to ININ by COFEPRIS (“Comisión Federal para la Protección contra Riesgos Sanitarios”: regulatory authority in Mexico), (c) the complete preclinical studies of ^99m^Tc-CXCR4-L, and (d) the basis of “proof-of-concept” and microdosing studies.

### 2.4. Acquisition of Images

^99m^Tc-CXCR4-L images in volunteers were obtained with a Symbia TruePoint dual-head gamma camera (SPECT/CT, Siemens), with high-resolution and low-energy collimators. The established parameters were velocity: 12 cm/min; matrix size: 256 × 1024 pixels; window: 20% symmetric at 140 keV; and scatter corrections: dual-energy window with simultaneous acquisition at 119 keV (20% width). Transmission factors to obtain the body (abdomen and chest) attenuation were calculated by using the *I*/*I*_0_ counting rate, with (*I*) and without (*I*_0_) the patient of a ^99m^Tc-filled flood source (555 MBq). Anterior and posterior scintigraphy of the whole body was obtained at 0.3, 1, 2, 4, 6, and 24 h after radiopharmaceutical administration.

In patients, preoperative (15 ± 5 d before surgery) cerebral SPECT (Siemens E. Cam Signature double detector) images were acquired at 3 h after ^99m^Tc-CXCR4-L (0.74 GBq) administration using a 128 × 128 matrix, window centered on 140 keV, with scattering correction, 360-degree rotation, 128 images of 20 s, and a total duration of approximately 21 min.

### 2.5. Image Analysis

Visual and semiquantitative analyses were performed by two physicians (specialized in nuclear medicine and molecular imaging) with an experience of >9 years in the use of the Siemens Syngo Acquisition Workplace equipment workstation with processing software for volumetric analysis. The tumor/background (*T*/*B*max) uptake ratio was calculated by quantifying the number of maximum counts obtained by delimiting volumetric regions (3D) of interest (VOI) with the isocontour around the entire tumor area (*T*) and in the contralateral brain region (*B*).

### 2.6. Biokinetic Evaluation

Images obtained in the DICOM (Digital Imaging and Communication in Medicine) format were processed using ImageJ Software (V1.51i, in Java, Image Processing and Analysis) for scattering correction by using the dual-energy window method. By using the transmission factors (*I*/*I*_0_) experimentally calculated as described above, regions of interest of source organs (liver, bladder, heart, spleen, intestine, lungs, kidneys, and whole-body) were corrected by attenuation. The activity in each source organ was divided by the initial whole-body (WB) activity (100% of injected activity) to determine the injected activity fraction (*IA*):

(1) $$\begin{array}{c} \%IA_{\text{source organ}}=\frac{A_{\text{source organ}}}{A_{\text{WB at the first image acquisition}}}\times 100. \end{array}$$

Technetium-99m time-activity curves were built from the image sequence in each organ. As the heart does not overexpress CXCR4, its activity was considered as having blood activity kinetics. The % *IA* data at different times were used in the OLINDA/EXM code to calculate the total number of disintegrations (*N*, MBq^.^h/MBq). The GI tract model (ICRP 30) included in the code was employed for the excretion model considering an activity fraction of 0.061–0.027 entering the small intestine, as images showed that 4.42 ± 1.69% of the ^99m^Tc-CXCR4-L injected activity is excreted to the intestine at 20 min after administration (Tables S1–S6, supplementary material). % *IA* in urine (bladder activity) was also considered as excretion data in the OLINDA code.

### 2.7. ^99m^Tc-CXCR4-L Absorbed Dose

Equivalent doses were evaluated according to general equation (2), as previously reported [15]:

(2) $$\begin{array}{c} D_{\text{target}\leftarrow \text{source}}=\sum_{\text{source}}N_{\text{source}}\times DF_{\text{target}\leftarrow \text{source}}, \end{array}$$

where *D*_target←source_ is the radiation absorbed, *N*_source_ is the total number of disintegrations, and *DF*_target←source_ is the absorbed dose per nuclear transition. The equivalent and effective radiation doses were obtained by using the experimental *N* values in the OLINDA/EXM code [15].

### 2.8. Tumor Tissue Samples

All patients underwent total or partial tumor resection. Histopathology was the gold standard to verify the diagnosis as well as to determine the presence of viable tumor tissue. The histopathological reports were collected by the Pathology Department of the “Hospital de Especialidades of CMN Siglo XXI, IMSS,” which were interpreted by a certified and experienced pathologist according to the World Health Organization (WHO) classification for tumors of the central nervous system (2016): high-grade gliomas (HGG) III-IV, low-grade gliomas (LGG) I-II, and metastasis.

### 2.9. Immunohistochemical Studies

Immunohistochemical staining of tumor specimens was carried out by using a human anti-CXCR4 monoclonal antibody (1 : 1000 dilution, MAB172, R&D Systems) with BOND equipment (Leica Biosystems). The analysis was performed double-blind by two pathologists, which selected five random fields for each tumor specimen under an optical microscope (magnification 200x). Gastric cancer tissue was used as an internal positive control for the expression of CXCR4.

### 2.10. Statistical Analysis

To analyze the relationship between the pathological grade (WHO classification) and the brain tumor uptake evaluated by SPECT (*T*/*B*max ratio), nonparametric tests were used.

## 3. Results and Discussion

**Figure 1:**
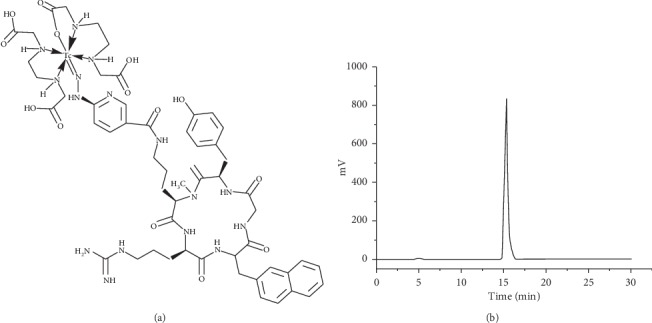
(a) Schematic structure of ^99m^Tc-CXCR4-L. (b) Reversed-phase radio-HPLC analysis of ^99m^Tc-CXCR4-L obtained from the freeze-dried kit before injection to patients with radiochemical purity >97%.

The radiochemical purity of ^99m^Tc-CXCR4-L (Figure 1), obtained from the freeze-dried kits, was 98 ± 1%, as obtained by HPLC analyses. The average molar activity was 13 GBq/*μ*mol before injection to patients.

None of the healthy volunteers reported adverse reactions such as bradycardia, itching, hives, vomiting, nausea, flushing, bronchospasm, dyspnea, coughing, chills, decreased blood pressure, or dizziness after ^99m^Tc-CXCR4-L administration.

The ^99m^Tc-CXCR4-L blood activity biokinetic model is shown in the following equation:

(3) $$\begin{array}{c} A\left(t\right)=95.2e^{-51.01t}+3.87e^{-3.41t}+0.927e^{-0.34t}. \end{array}$$

**Figure 2:**
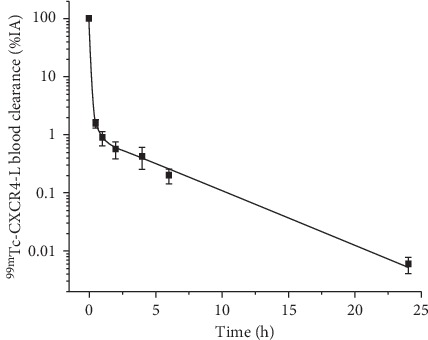
^99m^Tc-CXCR4-L blood clearance from healthy volunteers with *T*_1/2_*α* = 0.81 min, *T*_1/2_*β* = 12.19 min, and *T*_1/2_*γ* = 2.03 h.

**Figure 3:**
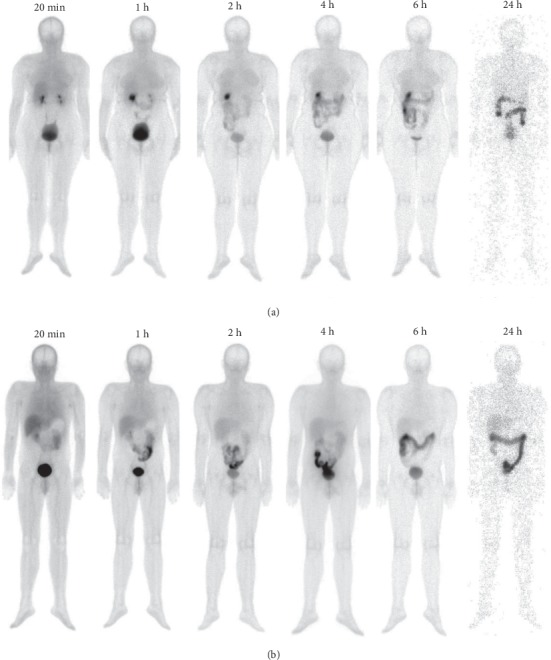
Whole-body images of two healthy volunteers, a female and a male, at 20 min, 1 h, 2 h, 4 h, 6 h, and 24 h after ^99m^Tc-CXCR4-L administration (0.37 GBq).

The half-life value was 0.0136 h (0.81 min) for the fast component (*T*_1/2_*α* = ln 2/51.01), 0.203 h (12.19 min) for the first slow component (*T*_1/2_*β* = ln 2/3.41), and 2.03 h for the second slow component (*T*_1/2_γ = ln 2/0.34) (Figure 2). The activity was rapidly eliminated by kidneys and the hepatobiliary system (Figure 3). The activity in the kidneys at 20 min was 4.28 ± 1.43%, and after 24 h, it decreased to 0.10 ± 0.07%. Twenty-four hours after the administration of ^99m^Tc-CXCR4-L, the total activity excreted from the whole body was 99.18 ± 0.24% (Supplementary Material, Tables S1–S6).

The total disintegrations that occurred in each organ are shown in Table 1, and the equivalent doses of ^99m^Tc-CXCR4-L for the main source organs are shown in Table 2. Due to the fast radiopharmaceutical elimination, the effective dose of ^99m^Tc-CXCR4-L (2.67–3.14 mSv/740 MBq) is in the same order of magnitude than that reported for other ^99m^Tc peptides, such as ^99m^Tc-RGD (6.1 mSv/740 MBq) or ^99m^Tc-iPSMA (3.42 mSv/740 MBq) [16, 17], and below 10 mSv, in agreement with the recommendation of the World Health Organization [18].

**Figure 4:**
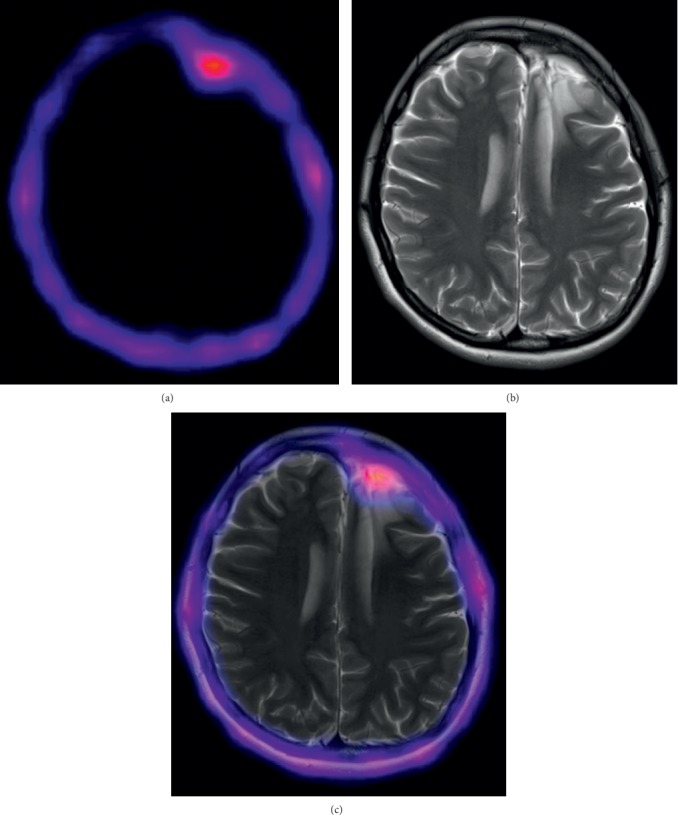
^99m^Tc-CXCR4-L SPECT (a), MRI (b), and SPECT/MRI (c) of a diffuse astrocytoma NOS (tumor recurrence) with *T*/*B*max 3.9 (patient 7).

**Figure 5:**
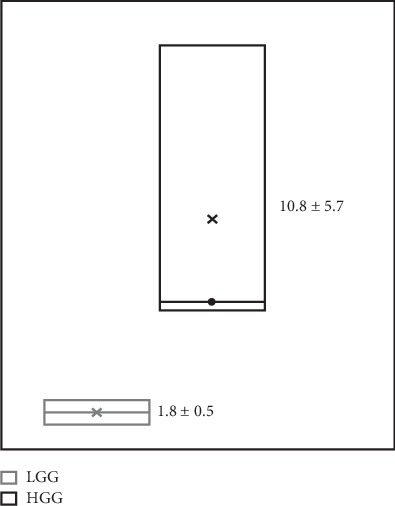
Correlation of the tumor/background uptake ratios (*T*/*B*max), evaluated by SPECT (^99m^Tc CXCR4-L), and the WHO pathological grade: high-grade gliomas (HGG) III-IV and low-grade gliomas (LGG) I-II (Spearman *r*_s_ = 0.94, *p* < 0.003).

Table 3 shows the *T*/*B*max ratios calculated using SPECT images from patients. Histopathological studies confirmed 3 HGG (1 gliosarcoma and 2 GBM), 2 LGG (oligodendroglioma), 1 metastasis (poorly differentiated carcinoma), 1 recurrent glioma (astrocytoma II), and 2 reactive glioses (see images in the Supplementary Material section). Among the 6 cases with an undetermined brain tumor, SPECT was positive in 3 HGG (*T*/*B*max 10.8 ± 5.7), 2 LGG (*T*/*B*max 1.8 ± 0.5), and 1 brain metastasis (*T*/*B*max 5.5). Of the patients with suspicion of tumor recurrence, SPECT was positive in one (diffuse astrocytoma II) with *T*/*B*max 3.9 (Figure 4), while for the rest, the result was negative, with a histological report of reactive gliosis. *T*/*B*max significantly correlated with the pathological grade (WHO classification) (Spearman *r*_s_ = 0.94, *p* < 0.003) (Figure 5).

**Figure 6:**
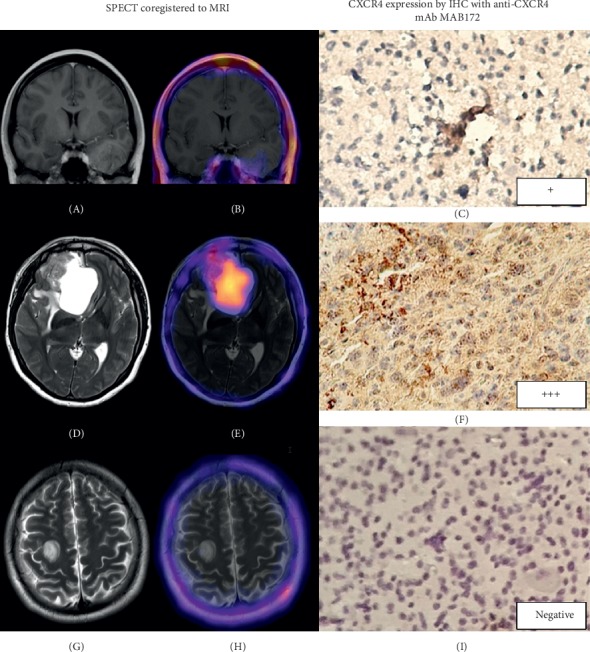
Follow-up of patients with suspected glioma by using magnetic resonance imaging (MRI) of the brain (A, D, and G), correlated with the lesion-specific uptake of ^99m^Tc-CXCR4-L SPECT/MRI (B, E, and H), and with the immunohistochemical detection of CXCR4 expression (C, F, and I). MRI of patient 1 with low-grade glioma (A) has no contrast enhancement and low radiotracer uptake (B); MRI of patient 4 with high-grade glioma (D) shows a hyperintense lesion in the frontal lobe and high radiotracer uptake (E); MRI of patient 9 (G) shows hyperintense areas in the subcortical location within the insulas and a rounded lesion in the right cerebral hemisphere negative for ^99m^Tc-CXCR4-L uptake (H), which suggests a diagnosis of gliosis. The biopsy of patient 1 with low-grade glioma (C) shows diffuse cytoplasmic CXCR4 protein immunostaining (+), while the biopsy of patient 4 with high-grade glioma (F) presents intense recognition of CXCR4 in the cytoplasmic and nuclear cell compartments (+++). Gliosis tissue of patient 9 (I) showed no HRP (horseradish peroxidase) staining, indicating the absence of CXCR4 cell expression, which correlates with the negative ^99m^Tc-CXCR4-L uptake.

Immunohistochemical results corroborated that CXCR4 was highly expressed in HGG, whereas its expression in LGG was very low and absent in reactive gliosis (Figure 6). As it is known, CXCR4 is overexpressed in various tumors, including HGG and brain metastasis, involved in the tumor growth, angiogenesis, recurrence, resistance to therapy, shorter survival, and poor conditioning prognosis [6]. In agreement with the results of this research, it has been reported that primary brain tumor cell lines have high concentrations of CXCR4 compared to the normal brain parenchyma, demonstrating CXCR4 overexpression of up to 81% in glioblastoma tumor tissues [19, 20]. In this context, Lapa et al. [7] used ^68^Ga-Pentixafor for glioblastoma PET imaging in 15 patients and compared it with ^18^F-FET (O-(2-^18^F-fluoroethyl)-L-tyrosine). They reported higher tumor/background ratios in the ^68^Ga images compared to those of ^18^F, as well as good retention in the tumor lesion. In this research, the use of SPECT with ^99m^Tc-CXCR4-L in patients with suspected brain tumors demonstrated a specific tumor uptake, which allowed adequate discrimination between tumor recurrence and gliosis. Likewise, the absence of intraparenchymal concentration permitted to obtain images for clinical use with appropriate *T*/*B*max ratios (HGG 10.8 ± 5.7, LGG 1.8 ± 0.5, and cerebral metastasis 5.5). The immunohistochemical confirmation of higher CXCR4 expression in patients with HGG with regard to those with LGG corroborated the directly proportional relationship between the grade of differentiation and the expression of CXCR4. This “proof-of-concept” research showed the potential utility of ^99m^Tc-CXCR4-L in the specific diagnosis and prognosis of brain tumors.

## 4. Conclusions

The equivalent and effective radiation doses of ^99m^Tc-CXCR4-L in humans are comparable to those from most diagnostic studies of ^99m^Tc. This preliminary study warrants further clinical studies in order to establish the usefulness of ^99m^Tc-CXCR4-L in the diagnosis, prognosis, prediction of therapeutic response, discrimination between recurrence, and reactive gliosis, as well as in the selection of patients who could benefit from targeted anti-CXCR4 therapies.

## Figures and Tables

**Table 1:** Average total number of disintegrations (*N*) of ^99m^Tc-CXCR4-L in source organs calculated from six healthy volunteers (3 females and 3 males).

Target organ	*N* (mean ± SD) (MBq·h/MBq)	
	Females	Males
Breasts	(1.39 ± 0.30) *E* − 01	
Gallbladder content	(3.07 ± 1.30) *E* − 02	(1.33 ± 0.09) *E* − 02
LLI content	(6.46 ± 1.27) *E* − 02	(7.27 ± 0.40) *E* − 02
SI content	(1.01 ± 0.20) *E* − 01	(1.14 ± 0.06) *E* − 01
ULI content	(1.32 ± 0.26) *E* − 01	(1.48 ± 0.08) *E* − 01
Heart content	(2.96 ± 0.18) *E* − 02	(3.68 ± 0.75) *E* − 02
Kidneys	(1.01 ± 0.20) *E* − 01	(1.46 ± 0.20) *E* − 01
Liver	(1.23 ± 0.41) *E* − 01	(1.66 ± 0.25) *E* − 01
Lungs	(1.70 ± 0.38) *E* − 01	(2.41 ± 0.40) *E* − 01
Testes		(2.53 ± 0.09) *E* − 02
Urinary bladder	(2.48 ± 0.40) *E* − 01	(1.70 ± 0.45) *E* − 01
Remainder of the body	1.13 ± 0.08	1.83 ± 0.04

**Table 2:** Average equivalent and effective doses of ^99m^Tc-CXCR4-L calculated from six healthy volunteers (3 females and 3 males).

Target organ	Equivalent doses (mean ± SD) (mSv/MBq)	
	Females	Males
Adrenals	(8.93 ± 1.31) *E* − 06	(8.38 ± 2.27) *E* − 06
Brain	(3.32 ± 0.25) *E* − 06	(3.54 ± 0.96) *E* − 06
Breasts	(3.02 ± 0.64) *E* − 04	(4.70 ± 0.26) *E* − 05
Gallbladder wall	(1.59 ± 0.48) *E* − 04	(1.00 ± 0.04) *E* − 04
LLI wall	(6.10 ± 0.64) *E* − 04	(6.10 ± 0.30) *E* − 04
Small intestine	(2.05 ± 0.21) *E* − 05	(1.71 ± 0.50) *E* − 05
Stomach wall	(1.87 ± 0.14) *E* − 04	(2.01 ± 0.47) *E* − 04
ULI wall	(3.30 ± 0.45) *E* − 05	(2.74 ± 0.80) *E* − 05
Heart wall	(5.26 ± 0.64) *E* − 05	(5.57 ± 0.53) *E* − 05
Kidneys	(3.13 ± 0.57) *E* − 05	(1.03 ± 0.89) *E* − 04
Liver	(1.41 ± 0.36) *E* − 04	(1.46 ± 0.16) *E* − 04
Lungs	(4.37 ± 0.90) *E* − 04	(4.71 ± 0.57) *E* − 04
Muscle	(5.81 ± 0.46) *E* − 06	(5.41 ± 1.56) *E* − 06
Ovaries	(5.63 ± 0.16) *E* − 04	(0.00 ± 0.00) *E*+00
Pancreas	(9.13 ± 0.12) *E* − 06	(8.35 ± 2.29) *E* − 06
Red marrow	(1.46 ± 0.01) *E* − 04	(1.67 ± 0.05) *E* − 04
Osteogenic cells	(2.68 ± 0.22) *E* − 05	(3.18 ± 0.09) *E* − 05
Skin	(6.57 ± 0.59) *E* − 06	(7.67 ± 0.25) *E* − 06
Spleen	(7.35 ± 0.80) *E* − 06	(7.02 ± 1.84) *E* − 06
Testes	(0.00 ± 0.00) *E*+00	(1.75 ± 0.07) *E* − 03
Thymus	(6.22 ± 0.77) *E* − 06	(5.63 ± 1.70) *E* − 06
Thyroid	(3.88 ± 0.36) *E* − 05	(5.45 ± 0.16) *E* − 05
Urinary bladder wall	(7.76 ± 1.15) *E* − 04	(4.06 ± 0.90) *E* − 04
Uterus	(1.52 ± 0.06) *E* − 05	(1.15 ± 0.38) *E* − 05
Effective dose (mSv/MBq)	(3.59 ± 0.29) *E* − 03	(4.24 ± 0.21) *E* − 03

**Table 3:** Histopathological results (classification of the World Health Organization) and tumor/background uptake ratios (*T*/*B*max) evaluated with ^99m^Tc-CXCR4-L radiopharmaceutical in patients with suspected brain tumors.

Patient no.	Age	Reported disease	WHO grade	*T*/*B*max ratio
1	30	Oligodendroglioma NOS	II	1.3
2	44	Oligodendroglioma NOS	II	2.4
3	42	Glioblastoma NOS	IV	6.6
4	72	Glioblastoma NOS	IV	7
5	59	Gliosarcoma	IV	19
6	51	Metastasis of poorly differentiated malignancy	NA	5.5
7	25	Diffuse astrocytoma NOS (tumor recurrence)	II	3.9
8	25	Reactive gliosis (tumor recurrence)	NA	NA
9	34	Reactive gliosis (tumor recurrence)	NA	NA

## Data Availability

The data used to support the findings of this study are included within the article.

## Supplementary Materials

Supplementary Materials In the supplementary material section, the whole-body images of healthy volunteers, including the tables of corrected counts, the percentage of injected activity (% IA) per organ at different times, and OLINDA calculations are presented. The cerebral SPECT images of all patients involved in this study are also included. 1. Whole-body images of healthy subjects (volunteers), tables of corrected counts, and % *IA*. 2. Example of the dose assessment: healthy subject 3 (female). 3. Example of the dose assessment: healthy subject 4 (male).
