# Supplementary material for: 99mTc-CXCR4-L for Imaging of the Chemokine-4 Receptor Associated with Brain Tumor Invasiveness: Biokinetics, Radiation Dosimetry, and Proof of Concept in Humans
Source: Contrast Media Mol Imaging. 2020 Apr 27;2020:2525037. doi: 10.1155/2020/2525037 (PMC7201577; doi:10.1155/2020/2525037)
Supplement: Supplementary Materials — In the supplementary material section, the whole-body images of healthy volunteers, including the tables of corrected counts, the percentage of injected activity (% IA) per organ at different times, and OLINDA calculations are presented. The cerebral SPECT images of all patients involved in this study are also included. 1. Whole-body images of healthy subjects (volunteers), tables of corrected counts, and % IA. 2. Example of the dose assessment: healthy subject 3 (female). 3. Example of the dose assessment: healthy subject 4 (male). [file 2525037.f1.pdf]

Contrast Media & Molecular Imaging

SUPPLEMENTARY MATERIAL

^{99m}Tc-CXCR4-L for Imaging of the Chemokine-4 Receptor-Associated with Brain Tumor Invasiveness: Biokinetics, Radiation Dosimetry and Proof of Concept in Humans

Paola Vallejo-Armenta [1], Clara Santos-Cuevas[2], Juan Soto-Andonaegui[1], Rosa M. Villanueva-Pérez[1], Jorge I. González-Díaz[1], Francisco O. García-Pérez[1,3], Angélica Arrellano-Zarate[1], Myrna Luna-Gutiérrez[2], Erika Azorín-Vega[2], Blanca Ocampo-García[2], and Guillermina Ferro-Flores[2*]

[1]Departamento de Medicina Nuclear. Hospital de Especialidades del Centro Médico Nacional Siglo XXI, Ciudad de México, 06720, MEXICO.

[2]Departamento de Materiales Radiactivos. Instituto Nacional de Investigaciones Nucleares (ININ), Ocoyoacac, 52750, Estado de México, MEXICO.

[3]Departamento de Medicina Nuclear. Instituto Nacional de Cancerología, Ciudad de México, 14000, MEXICO.

*Correspondence should be addressed to Guillermina Ferro-Flores; guillermina.ferro@inin.gob.mx

1. Whole-body images of healthy subjects (volunteers), tables of corrected counts and % IA

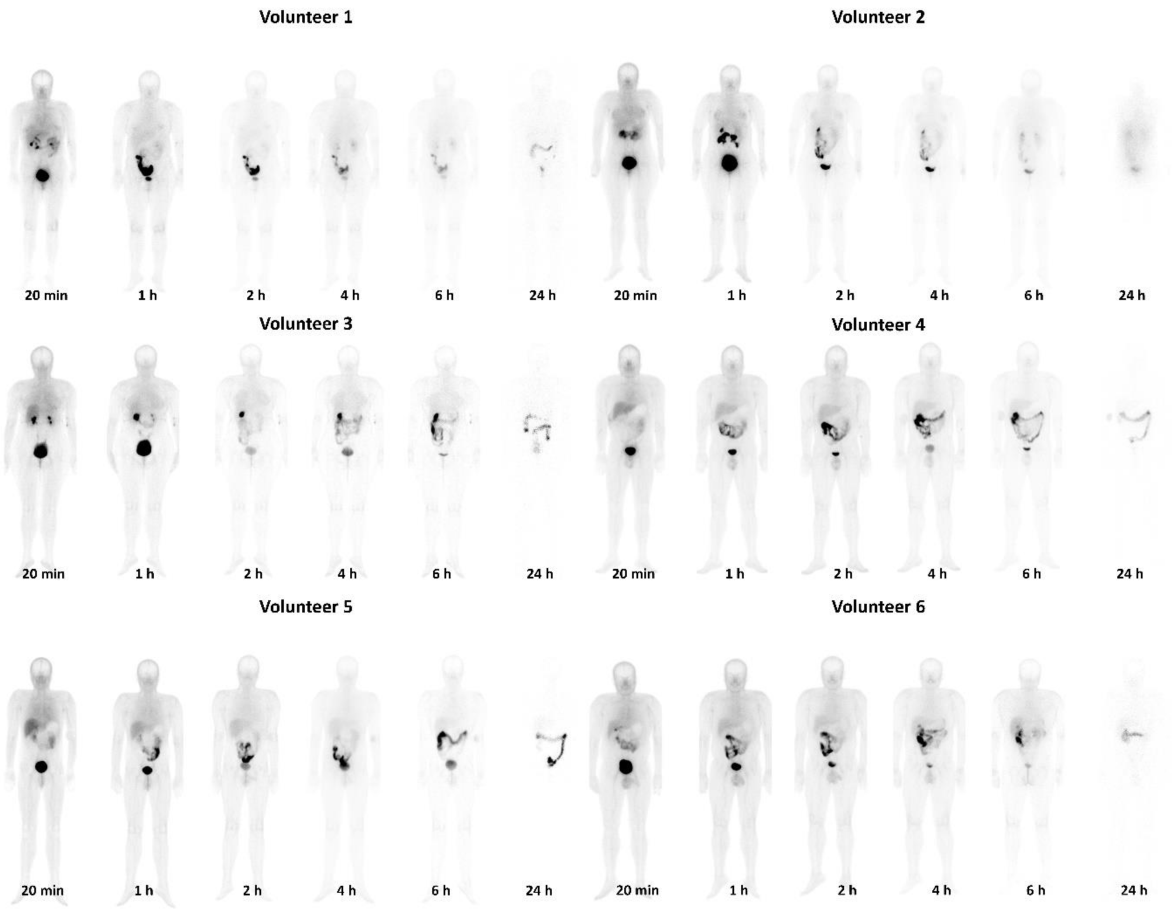

Fig. 1 Whole-body images of healthy subjects (volunteers) at 20 min, 1 h, 2h, 4 h, 6 h and 24 h after ^{99m}Tc-CXCR-4-L administration. The images depict the result of the geometric mean of anterior and posterior counts, according to the *conjugate views* method. Anterior and posterior images were previously corrected for attenuation and scattering.

Table 1. Corrected counts and %IA of source organs in healthy subject1

Healthy subject 1 (FEMALE)						
**ORGAN**	**CORRECTED COUNTS**					
	**20 min**	**1 h**	**2 h**	**4 h**	**6 h**	**24 h**
**WHOLE BODY**	2028039.98	1256007.95	590316.16	397972.54	232264.10	11647.24
**LIVER**	110917.54	46421.76	26148.01	27857.61	18710.04	457.49
**URINARY BLADDER**	233362.80	225730.78	26472.35	44036.84	14210.67	1832.64
**INTESTINE**	123410.44	146607.99	81455.18	20193.33	13149.05	2135.02
**GALLBLADDER**	12765.06	5630.41	4643.95	4050.19	2610.93	55.05
**LUNGS**	132544.07	68949.60	37104.75	36200.78	19454.84	513.65
**KIDNEYS**	122766.85	47106.14	22066.54	18518.77	10509.47	676.99
**BREAST**	130711.85	57375.25	31139.42	30410.70	16711.74	413.65
**HEART**	31929.31	13742.89	7523.81	6309.39	3340.23	95.76
**REMAINDER**	1129212.66	644443.14	353762.15	210394.92	133567.14	5466.98

Healthy subject 1 (FEMALE)						
**ORGAN**	% UPTAKE (%IA)					
	**20 min**	**1 h**	**2 h**	**4 h**	**6 h**	**24 h**
**WHOLE BODY**	100.00	61.93	29.11	19.62	11.45	0.57
**LIVER**	5.47	2.29	1.29	1.37	0.92	0.02
**URINARY BLADDER**	11.51	11.13	1.31	2.17	0.70	0.09
**INTESTINE**	6.10	7.23	4.02	1.00	0.65	0.11
**GALLBLADDER**	0.63	0.28	0.23	0.20	0.13	0.00
**LUNGS**	6.54	3.40	1.83	1.79	0.96	0.03
**KIDNEYS**	6.05	2.32	1.09	0.91	0.52	0.03
**BREAST**	6.45	2.83	1.54	1.50	0.82	0.02
**HEART**	1.57	0.68	0.37	0.31	0.16	0.00
**REMAINDER**	55.68	31.78	17.44	10.37	6.59	0.27

Table 2. Corrected counts and %IA of source organs in healthy subject2

Healthy subject 2 (FEMALE)						
**ORGAN**	CORRECTED COUNTS					
	**20 min**	**1 h**	**2 h**	**4 h**	**6 h**	**24 h**
**WHOLE BODY**	2035039.48	1212237.13	680621.64	496172.59	289575.45	14521.20
**LIVER**	98550.66	46237.93	35696.83	33383.89	22421.66	548.25
**URINARY BLADDER**	291650.05	313095.92	64206.14	43423.24	14012.67	1807.10
**INTESTINE**	59219.65	59490.43	51949.61	30684.03	19980.15	3244.19
**GALLBLADDER**	10885.23	10664.96	8957.97	8058.99	7830.21	106.42
**LUNGS**	165652.21	77855.01	53699.81	45706.01	24563.09	648.52
**KIDNEYS**	112666.87	68633.73	38126.03	29963.47	17004.38	1095.37
**BREAST**	157010.60	66964.97	46430.17	37744.70	20742.03	513.41
**HEART**	29107.10	12406.83	8380.82	6789.52	3594.41	103.05
**REMAINDER**	1084269.04	556887.36	373174.25	260418.74	159426.86	6454.88

Healthy subject 2 (FEMALE)						
**ORGAN**	% UPTAKE (%IA)					
	**20 min**	**1 h**	**2 h**	**4 h**	**6 h**	**24 h**
**WHOLE BODY**	100.00	59.57	33.45	24.38	14.23	0.71
**LIVER**	4.84	2.27	1.75	1.64	1.10	0.03
**URINARY BLADDER**	14.33	15.39	3.16	2.13	0.69	0.09
**INTESTINE**	2.91	2.92	2.55	1.51	0.98	0.16
**GALLBLADDER**	0.53	0.52	0.44	0.40	0.38	0.01
**LUNGS**	8.14	3.83	2.64	2.25	1.21	0.03
**KIDNEYS**	5.54	3.37	1.87	1.47	0.84	0.05
**BREAST**	7.72	3.29	2.28	1.85	1.02	0.03
**HEART**	1.43	0.61	0.41	0.33	0.18	0.01
**REMAINDER**	53.28	27.36	18.34	12.80	7.83	0.32

Table 3. Corrected counts and %IA of source organs in healthy subject3

Healthy subject 3 (FEMALE)						
**ORGAN**	CORRECTED COUNTS					
	**20 min**	**1 h**	**2 h**	**4 h**	**6 h**	**24 h**
**WHOLE BODY**	1885489.95	1494775.75	806172.37	549420.93	320652.16	16079.59
**LIVER**	133280.07	91522.48	68269.86	35961.67	24152.97	590.58
**URINARY BLADDER**	245848.31	343610.57	41280.23	39522.84	12754.01	1644.79
**INTESTINE**	50908.23	50415.00	40730.07	38084.32	24798.90	4026.62
**GALLBLADDER**	12502.92	29381.99	19554.07	14935.96	10744.07	146.02
**LUNGS**	18986.88	120600.82	82810.76	51038.13	27428.65	724.18
**KIDNEYS**	96124.55	60078.52	32497.08	38305.95	21738.77	1400.35
**BREAST**	150676.59	96922.60	67584.63	41668.73	22898.42	566.79
**HEART**	28803.21	18571.73	11392.74	6715.25	3555.10	101.92
**REMAINDER**	977437.80	683672.04	442052.93	283188.07	172581.27	6878.35

Healthy subject 3 (FEMALE)						
**ORGAN**	% UPTAKE (%IA)					
	**20 min**	**1 h**	**2 h**	**4 h**	**6 h**	**24 h**
**WHOLE BODY**	100.00	79.28	42.76	29.14	17.01	0.85
**LIVER**	7.07	4.85	3.62	1.91	1.28	0.03
**URINARY BLADDER**	13.04	18.22	2.19	2.10	0.68	0.09
**INTESTINE**	2.70	2.67	2.16	2.02	1.32	0.21
**GALLBLADDER**	0.66	1.56	1.04	0.79	0.57	0.01
**LUNGS**	1.07	6.40	4.39	2.71	1.45	0.04
**KIDNEYS**	5.10	3.19	1.72	2.03	1.15	0.07
**BREAST**	7.99	5.14	3.58	2.21	1.21	0.03
**HEART**	1.53	0.98	0.60	0.36	0.19	0.01
**REMAINDER**	51.84	36.26	23.45	15.02	9.15	0.36

Table 4. Corrected counts and %IA of source organs in healthy subject4

Healthy subject 4 (MALE)						
**ORGAN**	CORRECTED COUNTS					
	**20 min**	**1 h**	**2 h**	**4 h**	**6 h**	**24 h**
**WHOLE BODY**	3129097.28	2294855.64	1633927.01	1051893.01	675708.08	35254.54
**LIVER**	269909.53	161066.22	102766.71	61075.81	41334.81	1404.07
**URINARY BLADDER**	169673.7	94004.02	49904.94	30132.68	20055.20	572.76
**INTESTINE**	92308.37	130435.01	99333.21	72910.70	42310.01	3754.90
**GALLBLADDER**	18058.07	10775.94	6881.05	4825.02	3224.94	896.01
**LUNGS**	85024.93	179133.18	112032.10	60594.14	35401.86	896.01
**KIDNEYS**	122034.79	75552.96	105346.27	79708.79	77627.14	6794.20
**TESTES**	25675.92	23497.17	15906.16	9882.28	5640.17	143.02
**HEART**	39746.11	24001.27	15057.27	8353.78	4820.16	109.95
**REMAINDER**	2306665.86	1596389.87	1126699.30	724409.81	445293.78	20683.62

Healthy subject 4 (MALE)						
**ORGAN**	% UPTAKE (%IA)					
	**20 min**	**1 h**	**2 h**	**4 h**	**6 h**	**24 h**
**WHOLE BODY**	100.00	73.34	52.22	33.62	21.59	1.02
**LIVER**	8.63	5.15	3.28	1.95	1.32	0.04
**URINARY BLADDER**	5.42	3.00	1.59	0.96	0.64	0.02
**INTESTINE**	2.95	4.17	3.17	2.33	1.35	0.12
**GALLBLADDER**	0.58	0.34	0.22	0.15	0.10	0.03
**LUNGS**	2.72	5.72	3.58	1.94	1.13	0.03
**KIDNEYS**	3.90	2.41	3.37	2.55	2.48	0.22
**TESTES**	0.82	0.75	0.51	0.32	0.18	0.00
**HEART**	1.27	0.77	0.48	0.27	0.15	0.00
**REMAINDER**	67.32	51.02	36.01	23.15	14.23	0.66

Table 5. Corrected counts and %IA of source organs in healthy subject5

Healthy subject 5 (MALE)						
**ORGAN**	CORRECTED COUNTS					
	**20 min**	**1 h**	**2 h**	**4 h**	**6 h**	**24 h**
**WHOLE BODY**	3443105.95	2602445.19	2167193.81	1611579.61	787276.70	44484.1
**LIVER**	292038.91	178806.78	122371.65	99842.76	47593.80	1783.32
**URINARY BLADDER**	285471.52	174562.91	198952.99	92316.26	62515.49	10633.76
**INTESTINE**	201421.70	186775.34	201794.93	132519.79	6979.97	3830.98
**GALLBLADDER**	19862.05	12177.95	8405.02	7458.96	30482.00	748.90
**LUNGS**	325029.20	249739.91	153823.10	141096.90	74336.74	2352.75
**KIDNEYS**	105359.04	78160.01	69059.09	42376.87	75234.57	4313.67
**TESTES**	29252.17	26727.79	18196.27	16798.78	3133.96	132.93
**HEART**	69522.75	42674.11	26237.95	23438.27	9019.71	257.92
**REMAINDER**	2115099.99	1652820.38	1368352.80	1055731.03	477980.46	20429.87

Healthy subject 5 (MALE)						
**ORGAN**	% UPTAKE (%IA)					
	**20 min**	**1 h**	**2 h**	**4 h**	**6 h**	**24 h**
**WHOLE BODY**	100.00	75.58	62.94	46.81	22.87	1.18
**LIVER**	8.48	5.19	3.55	2.90	1.38	0.05
**URINARY BLADDER**	8.29	5.07	5.78	2.68	1.82	0.31
**INTESTINE**	5.85	5.42	5.86	3.85	0.20	0.11
**GALLBLADDER**	0.58	0.35	0.24	0.22	0.89	0.02
**LUNGS**	9.44	7.25	4.47	4.10	2.16	0.07
**KIDNEYS**	3.06	2.27	2.01	1.23	2.19	0.13
**TESTES**	0.85	0.78	0.53	0.49	0.09	0.00
**HEART**	2.02	1.24	0.76	0.68	0.26	0.01
**REMAINDER**	61.43	48.00	39.74	30.66	13.88	0.59

Table 6. Corrected counts and %IA of source organs in healthy subject6

Healthy subject 6 (MALE)						
**ORGAN**	CORRECTED COUNTS					
	**20 min**	**1 h**	**2 h**	**4 h**	**6 h**	**24 h**
**WHOLE BODY**	3129097.28	2255303.40	1799425.90	1024376.81	658032.41	20120.43
**LIVER**	165984.81	108960.89	87920.63	51429.52	34806.40	1082.38
**URINARY BLADDER**	390476.61	172096.65	76599.72	33078.65	22015.93	532.02
**INTESTINE**	188371.65	129122.76	116121.49	81420.01	47247.95	3841.75
**GALLBLADDER**	13035.98	6707.03	4882.71	5278.76	3528.21	89.20
**LUNGS**	249389.05	205490.86	158041.19	81527.16	47631.89	1461.20
**KIDNEYS**	93520.22	74135.04	71578.86	90908.40	88534.28	2170.84
**TESTES**	27385.59	22302.45	18319.15	10703.02	6108.60	147.67
**HEART**	49111.30	33370.17	24966.73	12957.81	7476.70	227.97
**REMAINDER**	1951930.88	1503117.55	1240995.43	657073.48	400682.45	10567.40

Healthy subject 6 (MALE)						
**ORGAN**	% UPTAKE (%IA)					
	**20 min**	**1 h**	**2 h**	**4 h**	**6 h**	**24 h**
**WHOLE BODY**	100.00	72.08	57.51	32.74	21.03	0.58
**LIVER**	5.30	3.48	2.81	1.64	1.11	0.03
**URINARY BLADDER**	12.48	5.50	2.45	1.06	0.70	0.02
**INTESTINE**	6.02	4.13	3.71	2.60	1.51	0.12
**GALLBLADDER**	0.42	0.21	0.16	0.17	0.11	0.00
**LUNGS**	7.97	6.57	5.05	2.61	1.52	0.05
**KIDNEYS**	2.99	2.37	2.29	2.91	2.83	0.07
**TESTES**	0.88	0.71	0.59	0.34	0.20	0.00
**HEART**	1.57	1.07	0.80	0.41	0.24	0.01
**REMAINDER**	62.38	48.04	39.66	21.00	12.81	0.34

3. Example of the dose assessment: Healthy subject 3 (female)

Table 7. Summary of %IA results, healthy subject 3

	20 min	1 h	2 h	4 h	6 h	24 h
**WHOLE BODY**	100.00	79.28	42.76	29.14	17.01	0.85
**LIVER**	7.07	4.85	3.62	1.91	1.28	0.03
**URINARY BLADDER**	13.04	18.22	2.19	2.10	0.68	0.09
**INTESTINE**	2.70	2.67	2.16	2.02	1.32	0.21
**GALLBLADDER**	0.66	1.56	1.04	0.79	0.57	0.01
**LUNGS**	10.07	6.40	4.39	2.71	1.45	0.04
**KIDNEYS**	5.10	3.19	1.72	2.03	1.15	0.07
**BREAST**	7.99	5.14	3.58	2.21	1.21	0.03
**HEART**	1.53	0.98	0.60	0.36	0.19	0.01
**REMAINDER**	51.84	36.26	23.45	15.02	9.15	0.36

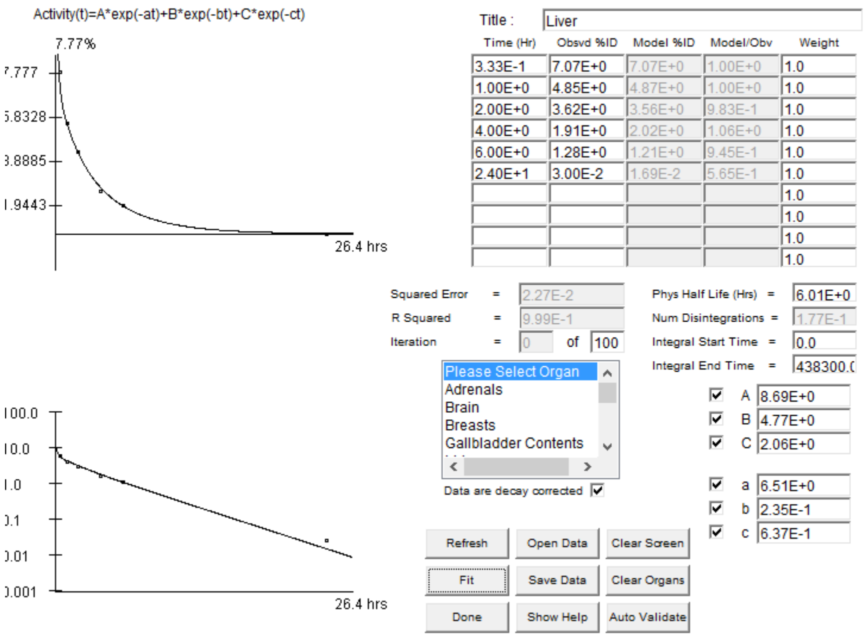

Fig. 2 Biokinetic model (e.g. liver) of healthy subject 3

From Fig. 1

$$q(t)_{ROI} = 8.69e^{-6.51(t)} + 4.77e^{-0.235(t)} + 2.06e^{-0.637(t)}$$

$$A(t)_{ROI} = 8.69e^{-(6.51+\lambda_{99mTc})(t)} + 4.77e^{-0.235+\lambda_{99mTc})(t)} + 2.06e^{-0.637+\lambda_{99mTc})(t)}$$

$$N = \int_{t=0}^{t=\infty} A(t)_{ROI}\, dt$$

$$N = \frac{8.69}{(6.51 + 0.1155)} + \frac{4.77}{(0.235 + 0.1155)} + \frac{2.06}{(0.637 + 0.1155)} = 17.7$$

As %Ia was used, the result is corrected to a fraction of the unit of activity administered to the patient by dividing N by 100

$$\boldsymbol{N = 0.177} \textbf{ MBq·h/MBq}$$

Table 8. Summary of N values, healthy subject 3

ORGAN	$N = \int_{t=0}^{t=\infty} A(t)_{ROI}\, dt$ (MBq·h/MBq)
**LIVER**	1.77E-1
**+URINARY BLADDER**	1.99E-1
***LLI CONTENT**	0.47E-1
***SI CONTENT**	0.74E-1
***ULI CONTENT**	0.96E-1
**GALLBLADDER**	2.83E-2
**LUNGS**	2.27E-1
**KIDNEYS**	1.59E-1
**BREAST**	1.82E-1
**HEART**	3.22E-2
**REMAINDER**	1.26E+0

*Note: The GI tract model (ICRP 30) included in the code was employed for the excretion model, considering an activity fraction of 0.027 (healthy subject 3) entering the small intestine, as images showed that 2.7% of the ^{99m}Tc-CXCR4-L injected activity is excreted to the intestine at 20 min after administration. +Considering voiding bladder model.

Table 9. Equivalent doses ($H_T = NxDF, mSv/MBq$) and effective dose ($H_E = \sum w_T H_T , mSv/MBq$) values, healthy subject 3

Target Organ	Alpha	Beta	Photon	Total	EDE Cont.	ED Cont.
**Adrenals**	0.00E+00	1.46E-04	1.66E-03	1.81E-03	0.00E+00	9.05E-06
**Brain**	0.00E+00	1.46E-04	4.52E-04	5.97E-04	0.00E+00	2.99E-06
**Breasts**	0.00E+00	3.32E-03	2.90E-03	6.22E-03	9.33E-04	3.11E-04
**Gallbladder wall**	0.00E+00	2.60E-03	2.94E-03	5.54E-03	3.32E-04	0.00E+00
**LLI wall**	0.00E+00	1.46E-04	1.28E-03	1.42E-03	0.00E+00	1.71E-04
**Small intestine**	0.00E+00	1.46E-04	1.63E-03	1.78E-03	0.00E+00	8.88E-06
**Stomach wall**	0.00E+00	1.46E-04	1.20E-03	1.35E-03	0.00E+00	1.62E-04
**ULI wall**	0.00E+00	2.75E-03	2.57E-03	5.31E-03	3.19E-04	2.66E-05
**Heart wall**	0.00E+00	4.86E-04	1.61E-03	2.10E-03	0.00E+00	0.00E+00

**Kidneys**	0.00E+00	3.80E-03	3.52E-03	7.32E-03	4.39E-04	3.66E-05
**Liver**	0.00E+00	8.31E-04	2.18E-03	3.01E-03	1.81E-04	1.50E-04
**Lungs**	0.00E+00	1.87E-03	1.90E-03	3.77E-03	4.52E-04	4.52E-04
**Muscle**	0.00E+00	1.46E-04	8.36E-04	9.81E-04	0.00E+00	4.91E-06
**Ovaries**	0.00E+00	1.46E-04	1.55E-03	1.69E-03	4.23E-04	3.38E-04
**Pancreas**	0.00E+00	1.46E-04	1.59E-03	1.73E-03	0.00E+00	8.67E-06
**Red marrow**	0.00E+00	8.44E-05	9.03E-04	9.88E-04	1.19E-04	1.19E-04
**Osteogenic cells**	0.00E+00	5.86E-04	1.55E-03	2.14E-03	6.42E-05	2.14E-05
**Skin**	0.00E+00	1.46E-04	4.35E-04	5.81E-04	0.00E+00	5.81E-06
**Spleen**	0.00E+00	1.46E-04	1.28E-03	1.42E-03	0.00E+00	7.11E-06
**Thymus**	0.00E+00	1.46E-04	1.06E-03	1.20E-03	0.00E+00	6.02E-06
**Thyroid**	0.00E+00	1.46E-04	5.72E-04	7.17E-04	2.15E-05	3.59E-05
**Urinary bladder wall**	0.00E+00	5.53E-03	5.81E-03	1.13E-02	6.80E-04	5.67E-04
**Uterus**	0.00E+00	1.46E-04	1.83E-03	1.98E-03	0.00E+00	9.88E-06
**Whole body**	0.00E+00	2.46E-04	9.52E-04	1.20E-03	0.00E+00	0.00E+00
**Effective dose (mSv/MBq)**						2.45E-03

3. Example of the dose assessment: Healthy subject 4 (male)

Table 10. Summary of %IA results, healthy subject 4

	**20 min**	**1 h**	**2 h**	**4 h**	**6 h**	**24 h**
**WHOLE BODY**	100.00	73.34	52.22	33.62	21.59	1.02
**LIVER**	8.63	5.15	3.28	1.95	1.32	0.04
**URINARY BLADDER**	5.42	3.00	1.59	0.96	0.64	0.02
**INTESTINE**	3.89	4.17	3.17	2.33	1.35	0.12
**GALLBLADDER**	0.58	0.34	0.22	0.15	0.10	0.03
**LUNGS**	9.11	5.72	3.58	1.94	1.13	0.03
**KIDNEYS**	2.97	2.41	3.37	2.55	2.48	0.22
**TESTES**	0.82	0.75	0.51	0.32	0.18	0.00
**HEART**	1.27	0.77	0.48	0.27	0.15	0.00
**REMAINDER**	67.32	51.02	36.01	23.15	14.23	0.66

healthy subject 4	
**ORGAN**	$$N = \int_{t=0}^{t=\infty} A(t)_{ROI}\, dt$$ **(MBq·h/MBq)**
**LIVER**	1.84E-1
**+URINARY BLADDER**	1.02E-1
***LLI CONTENT.**	0.68-1
***SI CONTENT**	1.07E-1

***ULI CONTENT**	1.39-1
**GALLBLADDER**	1.36E-2
**LUNGS**	1.87E-1
**KIDNEYS**	1.66E-1
**TESTES**	2.40E-2
**HEART**	2.56E-2
**REMAINDER**	1.81E+0

*Note: The GI tract model (ICRP 30) included in the code was employed for the excretion model, considering an activity fraction of 0.039 (healthy subject 4) entering the small intestine, as images showed that 3.89% of the ^{99m}Tc-CXCR4-L injected activity is excreted to the intestine at 20 min after administration. $^{+}$Considering voiding bladder model.

Table 11. Equivalent doses ($H_T = NxDF, mSv/MBq$) and effective dose ($H_E = \sum w_T H_T , mSv/MBq$) values, healthy subject 4

Target Organ	Alpha	Beta	Photon	Total	EDE Cont.	ED Cont.
**Adrenals**	0.00E+00	2.13E-04	1.77E-03	1.98E-03	0.00E+00	4.96E-06
**Brain**	0.00E+00	2.13E-04	6.18E-04	8.31E-04	0.00E+00	2.08E-06
**Gallbladder wall**	0.00E+00	1.27E-03	2.64E-03	3.91E-03	2.35E-04	0.00E+00
**LLI wall**	0.00E+00	2.13E-04	1.34E-03	1.55E-03	0.00E+00	1.87E-04
**Small intestine**	0.00E+00	2.13E-04	1.95E-03	2.16E-03	0.00E+00	5.41E-06
**Stomach wall**	0.00E+00	2.13E-04	1.33E-03	1.55E-03	0.00E+00	1.86E-04
**ULI wall**	0.00E+00	3.09E-03	2.97E-03	6.06E-03	3.63E-04	1.51E-05
**Heart wall**	0.00E+00	4.57E-04	1.46E-03	1.92E-03	0.00E+00	0.00E+00
**Kidneys**	0.00E+00	4.81E-03	4.10E-03	8.91E-03	5.35E-04	2.23E-04
**Liver**	0.00E+00	8.35E-04	2.20E-03	3.03E-03	1.82E-04	1.52E-04
**Lungs**	0.00E+00	1.62E-03	1.59E-03	3.21E-03	3.86E-04	3.86E-04
**Muscle**	0.00E+00	2.13E-04	9.44E-04	1.16E-03	0.00E+00	2.89E-06
**Pancreas**	0.00E+00	2.13E-04	1.71E-03	1.93E-03	0.00E+00	4.82E-06
**Red marrow**	0.00E+00	1.70E-04	1.06E-03	1.23E-03	1.48E-04	1.48E-04
**Osteogenic cells**	0.00E+00	1.10E-03	1.90E-03	3.00E-03	8.99E-05	3.00E-05
**Skin**	0.00E+00	2.13E-04	5.09E-04	7.22E-04	0.00E+00	7.22E-06
**Spleen**	0.00E+00	2.13E-04	1.42E-03	1.63E-03	0.00E+00	4.08E-06
**Testes**	0.00E+00	5.32E-03	2.88E-03	8.19E-03	2.05E-03	1.64E-03
**Thymus**	0.00E+00	2.13E-04	1.01E-03	1.23E-03	0.00E+00	3.07E-06
**Thyroid**	0.00E+00	2.13E-04	8.39E-04	1.05E-03	3.16E-05	5.26E-05
**Urinary badder Wall**	0.00E+00	2.31E-03	2.91E-03	5.22E-03	3.13E-04	2.61E-04
**Whole body**	0.00E+00	2.90E-04	1.05E-03	1.34E-03	0.00E+00	0.00E+00
**Effective dose (mSv/MBq)**						3.36E-03

4. Images of ^{99m}Tc-CXCR4-L uptake in patients with evidence of brain tumor injury

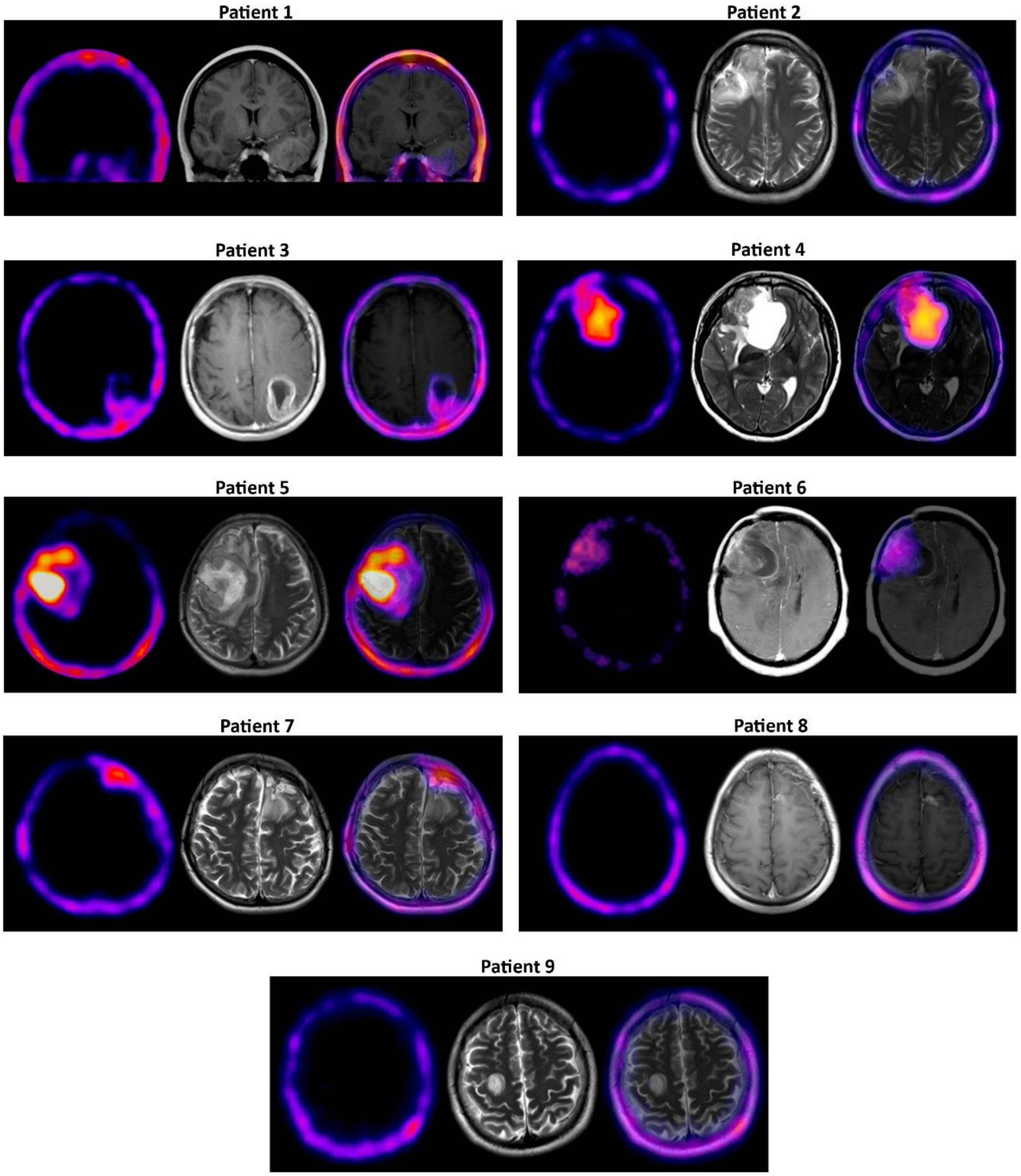

Fig. 3 Images show ^{99m}Tc-CXCR4-L SPECT (left), MRI (middle) and fused SPECT/MRI (right) of each patient that participated in the study.
